# The cost of evolved constitutive *lac* gene expression is usually, but not always, maintained during evolution of generalist populations

**DOI:** 10.1002/ece3.7994

**Published:** 2021-08-06

**Authors:** Kelly N. Phillips, Tim F. Cooper

**Affiliations:** ^1^ Department of Biology and Biochemistry University of Houston Houston Texas USA; ^2^ School of Natural and Computational Sciences Massey University Auckland New Zealand

**Keywords:** adaptation, compensation, experimental evolution, gene regulation

## Abstract

Beneficial mutations can become costly following an environmental change. Compensatory mutations can relieve these costs, while not affecting the selected function, so that the benefits are retained if the environment shifts back to be similar to the one in which the beneficial mutation was originally selected. Compensatory mutations have been extensively studied in the context of antibiotic resistance, responses to specific genetic perturbations, and in the determination of interacting gene network components. Few studies have focused on the role of compensatory mutations during more general adaptation, especially as the result of selection in fluctuating environments where adaptations to different environment components may often involve trade‐offs. We examine whether costs of a mutation in *lacI,* which deregulated the expression of the *lac* operon in evolving populations of *Escherichia coli* bacteria, were compensated. This mutation occurred in multiple replicate populations selected in environments that fluctuated between growth on lactose, where the mutation was beneficial, and on glucose, where it was deleterious. We found that compensation for the cost of the *lacI* mutation was rare, but, when it did occur, it did not negatively affect the selected benefit. Compensation was not more likely to occur in a particular evolution environment. Compensation has the potential to remove pleiotropic costs of adaptation, but its rarity indicates that the circumstances to bring about the phenomenon may be peculiar to each individual or impeded by other selected mutations.

## Graphical abstract

Beneficial mutations can become costly following an environmental change. We examined the cost of a specific regulatory mutation and the effectiveness of selection to reduce it, in populations of Escherichia coli bacteria. We found costs were significant, but could be reduced.

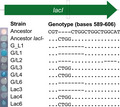

## 1 INTRODUCTION

Fluctuating environments pose several challenges to evolving populations. While some potential adaptations might confer benefits across all relevant selection regimes (Buckling et al., 2007, 2000; Kassen & Bell, 1998; Satterwhite & Cooper, 2015), others will confer benefits in some and costs in others (Bailey & Kassen, 2012; Jasmin & Kassen, 2007; Lee & Marx, 2012; McGee et al., 2015; Roemhild et al., 2015). Indeed, even if unconditionally beneficial mutations are initially available, they are likely to become less common over time (Martin & Lenormand, 2015; Satterwhite & Cooper, 2015; Schick et al., 2015). When a mutation that confers a benefit in one environment, and a cost in another, fixes in a population, it creates selection for subsequent mutations that compensate for that cost (Maisnier‐Patin et al., 2002; Moore et al., 2000; Moura de Sousa et al., 2017; Wood et al., 2013).

Compensatory mutations have long been used in molecular genetic studies as a tool to identify physical and genetic changes that can suppress the effects of a focal mutation, thereby identifying interacting components (Blank et al., 2014; Jarvik & Botstein, 1975; Kacar et al., 2017; Manson, 2000; Ponmani & Munavar, 2014; van Leeuwen et al., 2016). Increasingly, they are also recognized as being important in broadening the scope of evolutionary trajectories a population can follow (Szamecz et al., 2014; Zee et al., 2014), allowing adaptations to be selected that might otherwise prove to be evolutionary dead‐ends (Covert et al., 2013; Harrison et al., 2015), and influencing the ability of populations to simultaneously adapt to multiple environments (Melnyk et al., 2017).

Mutation interactions arising following selection in fluctuating environments have been extensively studied in the context of the evolution of antibiotic resistance. Antibiotic resistance mutations often confer a cost to bacteria in antibiotic‐free environments (Moura de Sousa et al., 2017; Nilsson et al., 2003; Rozen et al., 2007). This cost generates selection for subsequent compensatory mutations that relieve the cost (Björkman et al., 1998, 2000; Levin et al., 2000; Nagaev et al., 2001). Compensation allows resistance mutations to be maintained when they would normally be selected against, influencing short‐term evolutionary outcomes and perhaps longer‐term potential. Similar patterns of compensatory mutations depending on earlier resistance mutations for their benefit have been seen in studies of bacterial resistance to bacteriophage (Lenski, 1988; Wielgoss et al., 2016). Compensation during evolution has also been found to occur to overcome the loss of essential genes (Blank et al., 2014), the negative effects of synonymous (Knöppel et al., 2016), and gene deletion (Szamecz et al., 2014) mutations and to restore a social trait (Zee et al., 2014).

In contrast to studies that have focused on mutations that compensate for a specific genetic perturbation, few studies have examined compensation during more general adaptation to an environment, especially when this adaptation involves repeating rounds of selection in contrasting environments. This distinction might be important. Compensation to a specific genetic perturbation, such as deletion of a focal gene, is thought to generally act locally (Brandis et al., 2012; Filteau et al., 2015; Szamecz et al., 2014), although it can also arise in pathways unrelated to the perturbation (Blank et al., 2014). At least in experimentally evolving populations, adaptation often involves mutations in regulatory genes that are likely to have highly pleiotropic consequences (Cooper et al., 2003; Kurlandzka et al., 1991; MacLean et al., 2004; Rosenzweig et al., 1994). If costs of such pleiotropic mutations are revealed following an environmental change, it is not clear how subsequent compensatory mutations might affect fitness in the original environment. Indeed, it is easy to imagine that compensation causing a reduction in the cost of a focal mutation in a new environment might be associated with a reduction of the original benefit. In that case, reversion to the original environment might select for reversal of the effects of the compensatory mutation. This could occur through its direct reversion or through a second compensatory mutation, creating potentially complex patterns of environmentally dependent epistatic interactions between selected mutations.

The particular nature of the environmental fluctuations a population is exposed to is expected to play a major role in the selection of compensatory mutations. In a rapidly changing environment, mutations that increase in frequency are likely to confer a net benefit across the different environments (Buckling et al., 2007; Melnyk et al., 2017; Turner & Elena, 2000). With this limitation, such mutations can only confer at most relatively small costs in any one environmental component so that the strength of selection for compensation may be small (Poon & Chao, 2005, 2006). In a more slowly fluctuating environment, mutations selected in one component might fix before the population experiences a second component in which they might confer substantial costs (Bennett & Lenski, 2007; Kassen & Bell, 1998; Phillips et al., 2016). Such differential costs are consistent with the generally higher between‐environment trade‐offs seen in populations selected slowly compared to quickly fluctuating environments (Bono et al., 2017; Satterwhite & Cooper, 2015; Schick et al., 2015).

We examine compensation to an adaptive mutation selected in a series of experimentally evolved populations selected in environments that contained either lactose or glucose alone or a combination of lactose and glucose fluctuating daily or every 2,000 generations (Cooper & Lenski, 2010). Mutational inactivation of the LacI repressor was rapidly selected in many of the replicate bacterial populations that were selected in the presence of lactose (Quan et al., 2012). Loss of LacI causes the *lac* operon, a set of genes required for utilization of lactose, to be constitutively expressed (Markiewicz et al., 1994; Quan et al., 2012). When engineered into the ancestor of the evolution experiment, constitutive expression of the *lac* operon provided a benefit of ~9% during growth in lactose by shortening the lag time before resumption of growth following transfer into fresh medium (Quan et al., 2012). It also conferred a cost of ~3% in an environment containing glucose as the sole resource, probably due to some combination of the energetic cost of expressing unnecessary genes and toxicity of the LacY permease (Dekel & Alon, 2005; Eames & Kortemme, 2012; Quan et al., 2012; Stoebel et al., 2008). In populations selected in environments containing both lactose and glucose, this trade‐off in the effect of *lacI‐* mutations creates potential for subsequent mutations to provide a fitness benefit by compensating for the cost of the mutation in glucose.

We test whether evolved populations that fixed the *lacI‐* mutation, and therefore constitutively express the *lac* operon, evolved mechanisms that alleviate the cost of this expression during growth in glucose and, if so, whether this compensation comes at a cost of the benefit conferred by *lacI‐* in lactose. To do this, we isolated strains from populations evolved for 8,000 generations in lactose, glucose, and combinations of both fluctuating daily and every 2,000 generations. The *lacI‐* mutation was reverted in those strains that had substituted it, and its effect on fitness was determined. We found that the fitness cost of *lacI*‐ in glucose was variable, including, in one strain, becoming beneficial, but did not differ consistently between populations evolved in lactose only, where compensation is not expected to be selected, and in environments containing glucose, where it is. Similarly, strains varied in their relationship between the fitness benefit conferred by the *lacI*‐ mutation in lactose and costs in glucose, but this variation did not depend on their selection environment. Together, these results demonstrate the potential for the action of compensatory mutations to influence costs of adaptation but indicate that their effects may either be idiosyncratic or be overwhelmed by the effects of additionally selected mutations.

## 2 MATERIALS AND METHODS

### 2.1 Bacterial strains and strain construction

**FIGURE 1:**
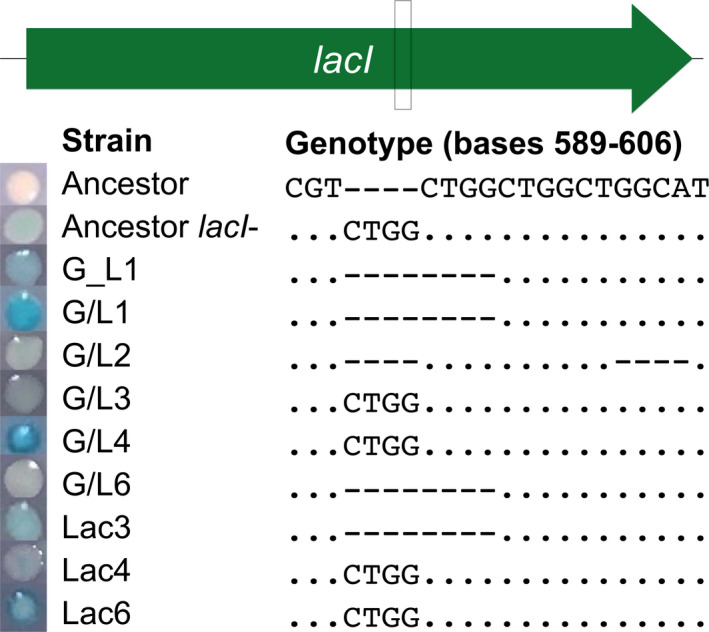
X‐gal phenotype and *lacI* mutations in focal clones. A representative colony of each clone grown on TGX indicator plates is shown at left. All mutations across evolved clones were either a 4 bp insertion or deletion frameshift mutation in a mutational hotspot region of *lacI* (box at top). Only the insertion is shown for the ancestor *lacI*‐, but the deletion was also assessed and had the same fitness effect and X‐gal phenotype

Bacterial strains were selected from replicate populations started with *Escherichia coli* B strains REL606 and REL607, which are isogenic except for a single base change in *araA*, which determines the ability to utilize arabinose, and a mutation in *recD* that also appears to be neutral (Tenaillon et al., 2016). Populations were evolved in Davis‐Mingioli (DM) minimal media supplemented with different combinations of glucose (175 μg/ml) and lactose (210 μg/ml) (Cooper & Lenski, 2010). The evolution environments included DM supplemented with lactose (Lac), daily fluctuations of glucose and lactose (G/L), or long‐term switching from glucose to lactose (G_L) or from lactose to glucose (L_G) every 2,000 generations. Each population was evolved for 8,000 generations, except for one G/L population, which needed to be restarted from an intermediate point during the evolution experiment and was evolved for 7,000 generations (G/L2). This difference has no consequence to the results reported here. Six replicate populations were evolved in each environment, and the replicate number is indicated by the number following the evolution environment designation. Clones with the *lacI‐* mutation were isolated from populations based on their growing as a blue colony on indicator plates that contained X‐gal (5‐bromo‐4‐chloro‐3‐indolyl‐beta‐D‐galactopyranoside) and glucose (TGX plates) (Figure 1; Quan et al., 2012). On TGX plates, the blue phenotype is indicative of a strain constitutively expressing the *lac* operon. A total of nine clones were chosen, one from each of three lactose‐only populations, a G_L population, and five G/L populations. In all clones, the *lacI* gene was amplified and sequenced to verify the presence of a mutant LacI repressor that we assume to be the cause of constitutive *lac* operon expression (Figure 1). Amplification was carried out using the primers: 5′‐GCGGAGCTGAATTACATTCC‐3′ (11‐F) and 5′‐GGGTGCCTAATGAGTGAGCT‐3′ (12‐R).

To determine the effect of substituted *lacI‐* mutations, we first constructed *lacI*+derivatives of our focal evolved strains. To do this, we PCR‐amplified the ancestral allele using primers 11‐F and 12‐R, ligated this product into pCR2.1 using a TA cloning kit (Invitrogen). The plasmid was used to transform TOP10F’ cells, which were blue/white screened to identify transformants having a plasmid insert. pCR2.1::*lacI*+plasmids were purified, and the *lacI*+fragment excised and cloned into pDS132 using enzymes SacI and XbaI. The resulting plasmid, pDS132::*lacI+,* was used to transform MFD*pir* cells (Ferrières et al., 2010). MFD*pir* (pDS132::*lacI* ^+^) was separately conjugated with each target evolved strain by mixing donor and recipient at a 1:2 ratio, respectively. Conjugation was carried out on LB agar plates supplemented with 2,6‐diaminopimelic acid (30 μM), which MFD*pir* needs for growth, for 3–4 hr at 37°C. The conjugation mixture was resuspended in 200 μl DM medium and plated onto minimal glucose agar (MG) supplemented with chloramphenicol (Cm; 20 μg/ml) to select for recipient strains with the pDS132 plasmid successfully integrated into the chromosome (Philippe et al., 2004). After overnight incubation, six colonies were restreaked onto MG +Cm agar, again incubated overnight, and then restreaked a second time to the same medium. A colony descended from each of the original six was resuspended in 500 μl DM base liquid media and plated onto sucrose plates supplemented with X‐gal to select for excision of the pDS132 plasmid (Philippe et al., 2004) and screen for clones that had retained the introduced *lacI*+allele. One blue colony and one white colony were selected from each plate and restreaked twice onto sucrose +X‐gal plates. White colonies indicate clones that successfully integrated the *lacI*+allele. Blue colonies, which retained the original *lacI‐* allele, were used as negative controls to test for the presence of additional mutations that occurred during the allele replacement process. All clones were tested for proper pDS132 plasmid excision by checking for chloramphenicol sensitivity. The *lacI*+insertion was verified by sequencing.

To evaluate the possibility of secondary mutations being inadvertently added during construction of *lacI*+strains, we measured the fitness of control strains that went through the conjugation process but did not retain the introduced *lacI+allele* in competition with an otherwise isogenic strain with a distinct neutral *ara* marker. The *ara* marker strains were constructed either by pairwise conjugations between the evolved strain and MFDpir (pDS132::*ara*‐) (same method as above except X‐gal was not used in the media) or by plating 100 μl of overnight LB cultures onto minimal arabinose agar (MA) and selecting for spontaneous *ara*+mutants. Indistinguishable fitness between competing strains was interpreted as indicating the absence of fitness‐effecting secondary mutations.

### 2.2 Genome sequencing

We report sequence of one evolved clone isolated from the G/L4 population. Genomic DNA was isolated and purified using the Wizard Genomic DNA Purification Kit (Promega). Libraries were created following the Nextera XT DNA Library Prep Kit protocol with Nextera XT Index Kit v2 adapters (Illumina). Libraries were pooled and sequenced on an Illumina NextSeq, producing 150 base‐pair, and the Breseq computational pipeline was used to align reads to the REL606 reference sequence and identify mutations (Deatherage & Barrick, 2014).

### 2.3 Fitness assays

Fitness effect of the *lacI*‐ mutation in each isolated strain was measured in glucose (175 μg/ml) and lactose (210 μg/ml)‐only environments. Cells were transferred from freezer stocks initially to LB medium and then, after overnight growth, to minimal medium supplemented with glucose or lactose as used in the particular competition assay. Strains were initially acclimated to the assay environment over two 24 hr transfer cycles with a 1:100 dilution occurring between each cycle. Preconditioned competitors were mixed at a 1:1 ratio by diluting each competing strain 1:200 directly into the assay environment. Competitions were carried out over two (lactose competitions) or four (glucose competitions) transfer cycles. A greater number of transfer cycles was used in glucose to be able to detect the small fitness differences between strains generally present in that environment. On the initial and final day of competitions, cells were plated onto TGX plates and incubated at 37°C for 18–20 hr in order to distinguish competing genotypes. Relative fitness effect of the *lacI*‐ mutation was determined based on the change in density of blue (*lacI*‐) and white (*lacI*+) colonies on TGX plates using the formula: ln(blue_2_ × 100 t/blue_0_)/ln(white_2_ × 100 t/white_0_), where subscripts indicate the time at which competitor density was estimated, and *t* accounts for transfer cycles during the competition (Lenski et al., 1991). Test competitions checking for additional mutations occurring during allele replacement procedures or selection of spontaneous *ara*+mutants were performed as described above, except competitors were distinguished by plating onto tetrazolium arabinose agar (TA).

### 2.4 Expression assays

Expression of the *lac* operon was measured using a GFP reporter construct controlled by the P*_lac_* promoter region including the O1 and O3 LacI operator sequences, and native primary CRP binding site (Quan et al., 2012). This reporter was previously cloned into a mini‐Tn*7* cassette in a suicide‐vector (Quan et al., 2012) and was introduced into target strains by tri‐parental conjugations between a target recipient evolved strain, a donor strain (MFD*pir* (pUC18R6KT::P*_lac_*‐GFP, kan^r^)), and a helper strain (MFD*pir* (pTSN2)) (Choi et al., 2005; Ferrières et al., 2010; Quan et al., 2012). Strains were mixed at a 3:1:1 ratio (recipient: donor: helper) on LB +DAP (30 μM) agar and incubated at 37°C for 3 hr. The conjugation mix was resuspended in DM medium and plated onto LB +kanamycin (Km; 60 μg/ml) + isopropyl β‐D‐1‐thiogalactopyranoside (IPTG; 1 mM) agar plates. Kanamycin selects for clones that successfully obtained the P*_lac_*‐GFP reporter while IPTG induces expression of the reporter allowing identification of clones that stably expressed GFP. After 24–36 hr of growth, six fluorescent colonies were restreaked onto LB +Km (60 μg/ml) plates. Restreaked colonies were streaked a second time on LB +Km + IPTG plates and then tested for absence of the delivery plasmid by spotting colonies on LB +Ap (100 μg/ml) agar. Insertion of the P*lac*‐GFP reporter into the *att*Tn7 site was confirmed by PCR (primers: 5′‐TAACAGCCAGCACCACGCCG‐3′ (120‐F) and 5′‐CGCGAATCCGATCTGGCGCT‐3′ (121‐R)). Transposition of the reporter into each recipient strain's *att*Tn*7* site allows consistent insertion of the reporter into the same region of the genome, minimizing divergent effects on reporter fitness and expression costs.

To measure P*_lac_*‐GFP reporter expression, reporter strains were grown overnight in LB broth from freezer stocks, diluted 1:10,000 in the assay environment, and allowed to grow for 24 hr. The following day cultures were diluted again 1:10,000 in the assay environment and grown in a VersaMax spectrophotometer (Molecular Dynamics, CA) until mid‐log phase (OD_450_ ~0.1–0.15) to allow measurement of a steady‐state level of P*_lac_*‐GFP reporter expression. P*_lac_*‐GFP expression was measured in an Accuri C6 flow cytometer (Becton Dickinson, NJ). Assay environments included DM+175 μg/ml glucose, DM+210 μg/ml lactose.

### 2.5 Statistical analyses

Data were analyzed using R (version 3.5.0; R Core Team, 2018). Dunnett's tests for one against many comparisons were performed using the glht function in the multcomp package. Where appropriate, experimental block was included in analyses as a random effect in mixed‐model ANOVA performed using the lmer function in the lme4 package. Fixed effects were tested for significance by comparing models fitted with and without the factor of interest using a chi‐square test.

## 3 RESULTS

### 3.1 Evolution of compensatory mutations

**FIGURE 2:**
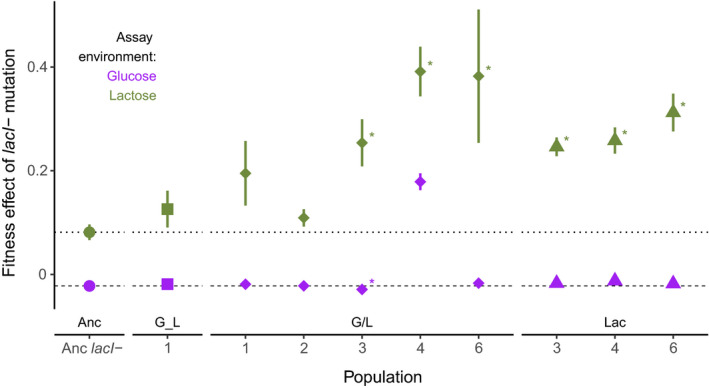
Fitness effect of *lacI*‐ in the ancestor and evolved genetic backgrounds measured in minimal glucose (purple symbols) and lactose (green symbols) environments. Asterisks indicate clones with significantly different *lacI*‐ fitness effect when compared to the effect in the ancestor (Dunnett's test, *p* < .05). Symbols and error bars indicate mean and standard error of at least 10 (in the glucose environment) or three (in the lactose environment) replicate fitness estimates. Note that error bars are smaller than symbols for many of the glucose fitness estimates. The dashed lines represent the fitness effect of *lacI*‐ in the ancestor in glucose (dashes) and lactose (dots). Evolution environments are shown directly above clone names which denotes the population a clone was selected from (“Anc” indicates the ancestor). In the glucose environment, one clone had a significantly higher fitness than the ancestor indicating that compensatory mutations are alleviating the cost of *lacI*‐ in glucose

In the ancestor to our evolution experiment, mutations that inactivate the LacI repressor, and lead to constitutive expression of the *lac* operon, confer a fitness cost of approximately 2.2% during growth in glucose (Quan et al., 2012). To determine whether this cost is compensated during evolution in environments containing both glucose and lactose resources, and, if so, whether this compensation differed depending on the presentation of the two resources, we reverted evolved *lacI‐* mutations in nine clones isolated from populations selected in lactose alone (Lac, three populations), long‐term switching of glucose and lactose (G_L, one population), or daily switching of glucose and lactose (G/L, five populations). We found that the cost of *lacI*‐ in glucose was significantly changed only in evolved clone G/L4 where the mutation had become beneficial (Figure 2; Dunnett's Test, *p* < .001). This result indicates the presence of sign epistasis, in that the *lacI‐* mutation changed from being deleterious in the ancestor to being beneficial in the evolved background. No difference in evolved *lacI‐* costs was detectable among other evolved clones (χ^2^ = 7.82, *df* = 8, *p* = .45).

### 3.2 Effect of environment on compensatory mutations

Although the cost of *lacI‐* was significantly changed in only one clone when compared to the ancestor, there may be changes in costs apparent when grouping clones based on their evolution environment. To test this, we compared the effect of the *lacI‐* mutation on fitness in glucose of each evolution environment group (strains evolved in Lac, G/L, or G_L). We expect clones evolved in the presence of fluctuations of glucose and lactose (i.e., G/L and G_L selection environments) to have a reduced fitness cost of the *lacI‐* mutation in glucose because compensation for the original cost would provide an advantage. By contrast, selection for compensatory mutations was expected to be reduced or absent in the lactose‐only environment. We found no significant difference in cost of the *lacI‐* mutation measured in the glucose environment among clones isolated from different evolution environments whether or not the outlier clone, G/L4, was included (ANOVA with G/L4: *F*_2,94_ = 1.68, *p* = .19; without G/L4: *F*_2,88_ = 0.98, *p* = .38).

### 3.3 Pleiotropic effect of *lacI*‐ compensation

**FIGURE 3:**
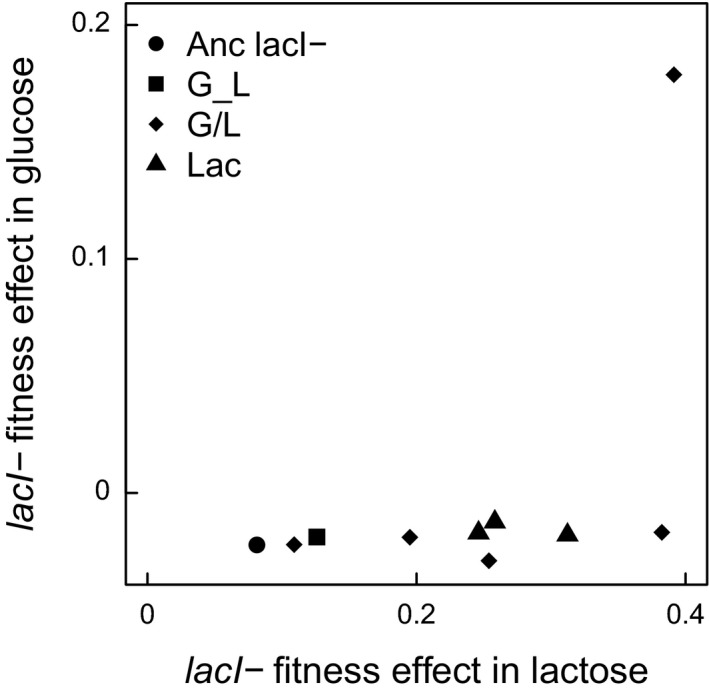
Fitness effect of *lacI*‐ in ancestor and evolved clones measured in glucose and lactose environments. Symbol shapes indicate the evolution environment or genotype of each clone. The ancestral measurements (circle) indicate the expectation if there were no epistatic interactions between *lacI*‐ and other mutations in the evolved background

Given that compensation for the cost of constitutive *lac* expression evidently can occur, one explanation for the low frequency at which it does occur is that it imposes a correlated cost in lactose. For example, it might be that compensation to constitutive expression of the *lac* operon involves a reduction in the maximum level of *lac* expression, perhaps reducing fitness in lactose and thereby causing compensation to be selectively disfavored. To test this possibility, we examined the fitness effect of *lacI*‐ mutations across glucose and lactose environments. To determine if there was any trend of a lower cost of *lacI*‐ in glucose corresponding to a lower benefit in lactose, we determined the relationship between the fitness effect of *lacI*‐ mutations in glucose and lactose across all strains. We found a marginally significant positive correlation between fitness in the two environments, indicating that a low cost of the *lacI‐* mutation in glucose was, if anything, associated with an increased benefit in lactose (Figure 3; Spearman's rank correlation, rho = 0.73, *p* = .021). The significance of the correlation is dependent on the G/L4 evolved clone that compensated for the *lacI‐* mutations cost in glucose. When that clone was omitted from the analysis, the correlation was no longer significant, though was still positive (rho = 0.63, *p* = .08). Focusing on the G/L4 clone revealed that the *lacI*‐ mutation is beneficial in glucose and its effect in lactose is significantly higher than in three of the evolved clones tested, as well as the ancestor (G_L1, G/L1, and G/L2; Dunnett's test, *p* < .05). Together, these results indicate that there is no trade‐off with fitness in lactose that limits selection for compensation of *lacI*‐ costs in glucose.

### 3.4 Mechanisms of lowered costs

**FIGURE 4:**
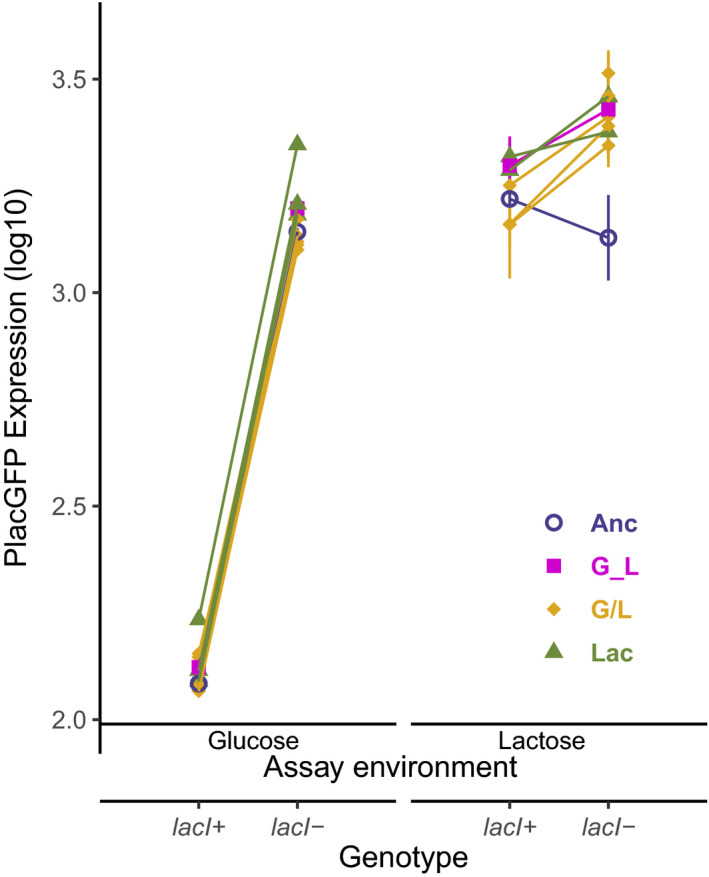
P*lac*‐GFP reporter expression of *lacI*+and *lacI*‐ clones in glucose and lactose. The symbols and colors indicate the genetic background and evolution environment of each clone. The X‐axis shows the genotype and assay environment that P*lac‐*GFP expression was measured in. Each measurement is the average of the median expression values of independent replicates (*n* = 14 in glucose and *n* = 4 in lactose). Error bars show standard error of the mean (note that error bars are generally covered by symbols in the glucose environment assays)

**FIGURE 5:**
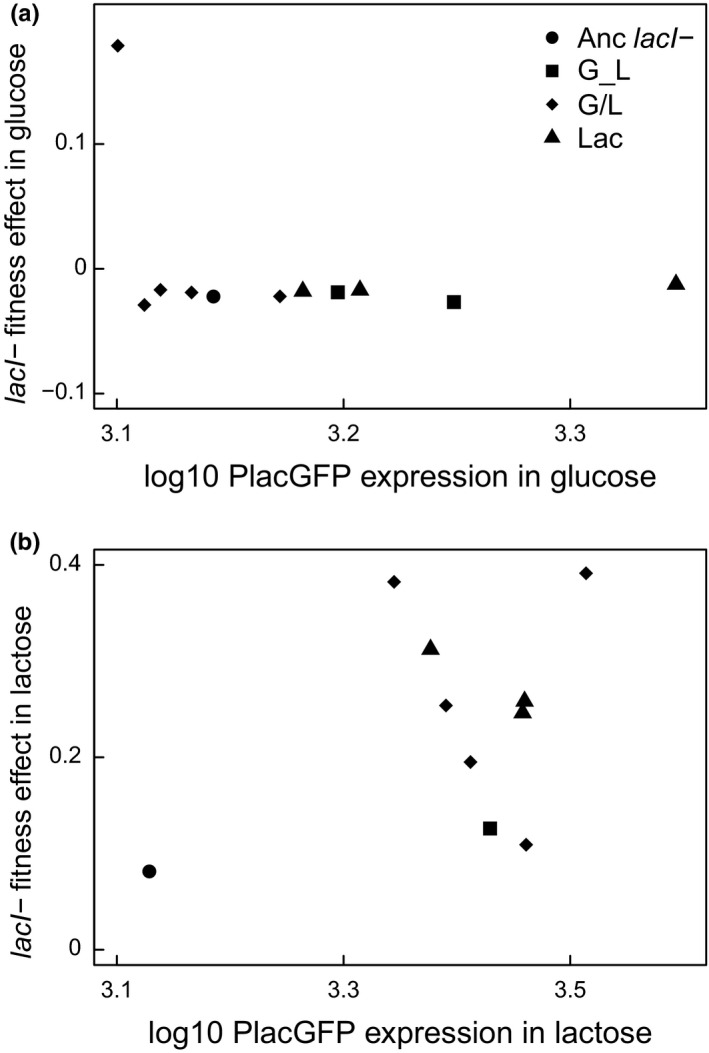
Relationship between *lac* expression and *lacI‐* fitness effect. Symbols indicate the mean fitness effect and *lac*‐reporter expression of the ancestor and evolved clones as indicated in panel a. (a) Expression and fitness estimates measured in the glucose environment. (b) Expression and fitness estimates measured in the lactose environment

To determine if *lac* operon expression is associated with changes in the fitness effects of the *lacI*‐ mutation, we measured *lac* operon expression in glucose using a reporter that is controlled by the promoter region, P*_lac,_* that drives expression of the *lac* operon (Figure 4). Expression of the *lac* operon contributes to the cost of constitutive expression, so we expected a negative relationship, such that clones that had higher *lac* operon expression would have lower fitness in the glucose environment (i.e., a higher cost; Dekel & Alon, 2005; Stoebel et al., 2008). In fact, there was no correlation (Figure 5a; Spearman's rank correlation, rho = 0.27, *p* = .49). This is especially surprising because half of the evolved strains had significantly higher *lac* expression in glucose than the ancestor *lacI*‐, so that an effect of *lac* expression on fitness could have been detected (Dunnett's test: G_L1, G/L4, Lac3, Lac4, Lac6 *p* < .05). That increased expression was not associated with any fitness cost might indicate the action of compensation to some portion of the cost that would otherwise be associated with increased *lac* expression. Alternatively, there could be a limit to the cost associated with constitutive *lac* operon expression (Eames & Kortemme, 2012), although the model most analogous to the situation prevailing in our experiments predicts exponentially increasing costs with increasing expression (Dekel & Alon, 2005).

All clones except G/L4 had similar *lacI‐* associated fitness costs when compared to the ancestor, but clones varied when it came to differences in expression compared to the *lacI*‐ ancestor. G/L4 had equal lowest *lac* expression in glucose (Dunnett's test, *p* < .05; except for G/L3, *p* = .87; and G/L6, *p* = .43), and all G/L clones had lower expression in glucose when compared to all Lac clones. Together, these results indicate that there was some differential evolution of *lac* expression based on environment, but that consequences do not consistently map to fitness effects: clones with similar expression levels in glucose have different *lacI‐* fitness effects (G/L4 compared to G/L3 and G/L6), and clones with similar *lacI‐* fitness effects have different expression (G/L compared to Lac clones). Evidently, reduced *lac* operon expression cannot explain all of the decreased costs in glucose and alleviation of the cost is dependent on other mutations in the evolved background.

Finally, our reporter strains allow us to address the relationship between *lac* expression and fitness in the lactose environment. We expected a positive correlation between *lac* expression and fitness if higher *lac* expression directly contributed to fitness in lactose. In fact, although all evolved clones had increased *lac* expression, changes were not correlated with fitness (Figure 5b; Spearman correlation, rho = −0.18, *p* = .64). Moreover, although the *lacI‐* mutation conferred one of the biggest benefits when added to the G/L4 clone, *lac* expression was not significantly different in this clone compared to other evolved clones (Dunnett's test, *p* > .05). These results suggest that the benefit of higher expression depends on the genetic background in which it occurs and that most of the changes in maximum *lac* expression are not caused by *lacI*‐ itself but by differences in the broader genetic background.

## 4 DISCUSSION

Few studies have focused on the influence of compensatory mutations during adaptation to a general environment rather than to a specific genetic perturbation. Our results demonstrate that compensation to deleterious consequences of an adaptive mutation is possible but is rare among evolved clones in this experiment. Of the nine clones tested, only one clone evolved a mechanism to alleviate the cost of constitutive *lac* operon expression in glucose (Figure 2). No differences in compensation were evident comparing groups of clones evolved in different environments, including some that were and some that were not expected to select for compensation.

Compensation of deleterious mutation effects has been observed in many systems and contexts—including compensation of costs due to antibiotic resistance, adaptation, and gene deletion (Björkman et al., 1998, 2000; Blank et al., 2014; Knöppel et al., 2016; Levin et al., 2000; Nagaev et al., 2001; Szamecz et al., 2014; Zee et al., 2014). A common theme is that compensation is readily selected, consistent with there being many available genetic mechanisms. For example, one study found 68% of single‐gene deletion slow growth mutants could be compensated to restore growth to near wild type within ~400 generations (Szamecz et al., 2014). Indeed, compensation to the costs of the *lacI‐* mutation apparent in glucose containing environments was possible in our experiment, but occurred in only one of the nine clones we examined. One explanation is that compensation is limited not by mutational opportunity but by selection for those mutations. Our populations evolved asexually so that beneficial mutations of large effect outcompete those of smaller effect (Gerrish & Lenski, 1998). Thus, even if they arise, small benefit compensatory mutations might be outcompeted until mutations of large effect are exhausted (Leon et al., 2018). Our populations exhibit higher fitness gains at early versus later time points (Satterwhite & Cooper, 2015); however, the latter gains may still be larger than the small ~2.2% cost of *lacI*‐ in glucose. Indeed, the G_L populations steadily increased in glucose fitness up to 6,000 generations so compensatory mutations may not be sufficiently competitive to be selected (Satterwhite & Cooper, 2015). By contrast, G/L populations had small or undetectable fitness change across glucose and lactose environments after 4,000 generations, which would have allowed selection of small benefit mutations and may be why only a G/L clone was suggested to have compensatory mutations (Satterwhite & Cooper, 2015).

Another possible explanation for the rarity of compensation is that multiple mutations are required to compensate the cost of *lacI*‐ in glucose without affecting its benefit in lactose (Poon et al., 2005). Multiple compensatory mutations will take longer to fix and will be rare because successful compensation can depend on the order in which each required mutation occurs (Gong et al., 2013), the presence/absence of other mutations in the genetic background (Lunzer et al., 2010; Shah et al., 2015), and a clone's fitness at each step relative to others in the population which can subject an intermediate clone to being purged by purifying selection (Gerrish & Lenski, 1998). Replacing *lacI*‐ with a functional LacI repressor in the G/L4 clone, in which compensation did occur, significantly reduces fitness in glucose and expression of the *lac* genes. In other words, this clone has somehow rewired the *lac* network such that *lac* operon expression is beneficial for growth in glucose. The basis of this rewiring is unknown but might depend on multiple mutations as is the case, for example, in the case of evolved citrate utilization selected in a population started from the same ancestor as the one used here (Blount et al., 2008, 2012; Quandt et al., 2015).

We consider that LacY is the best candidate *lac* gene to be involved in some new compensatory interaction. Not only is it the likely source of the cost of constitutive *lac* expression (Eames & Kortemme, 2012), but it can also support glucose uptake, though, as characterized, that requires a mutational change in the enzyme that did not occur in clone G/L4 (Gram & Brooker, 1992; King & Wilson, 1990; Sahin‐Tóth et al., 2001). Although glucose transport is not likely to limit growth at the concentration used in this experiment, it is possible that an alternative import mechanism could provide an advantage by reducing dependence on the phosphotransferase system (Ferenci, 1996; Jahreis et al., 2008; Postma et al., 1993). This system is a common mutational target in populations evolved from the same ancestor and selected in a glucose environment, suggesting it is not optimized for growth in the selective environment prevailing in our experiment (Woods et al., 2006). Sequencing of a clone isolated from the G/L4 population identified a mutated gene, *sohB,* that is a candidate for interacting with LacY and that was not mutated in the other populations we examined here or in lines that evolved only in glucose (Table S1). SohB is a peptidase active, along with LacY, in the cell periplasm (Baird et al., 1991). Although the function of SohB is not well characterized, it has been shown to be able to compensate for loss of other peptidases involved in periplasm protein recycling, suggesting a possibility of affecting LacY function.

An alternative mechanism of *lacI‐* compensation is a change in *lacI* regulation. This could occur in either of two ways. First, the 4 bp insertion frameshift mutation in *lacI*‐ of G/L4 results in a truncated 204 residue LacI repressor. Although nonfunctional at the *lac* operator, it is possible that the truncated LacI, which includes the DNA binding domain, interacts with another region in the genome to cause some beneficial effect that is lost when the ancestral *lacI* was restored (Platt et al., 1973). Second, an operator site for a different gene may have evolved to provide a benefit in the absence of LacI. The LacI repressor has similarity to other repressors in *E. coli* (Weickert & Adhya, 1992), and studies have shown that as few as two mutations are needed to increase the affinity of LacI to another operator (Daber & Lewis, 2009; Lehming et al., 1987, 1990; Salinas et al., 2005). We note, however, that the sequenced G/L4 clone does not have mutations in recognized *lacI* family operator sites, so a change in sites bound by LacI is not predicted (Table S1).

In summary, compensation was rare and did not occur based on specific fluctuations of glucose and lactose. In the clone in which compensation of *lacI*‐ did occur, *lac* operon expression was reduced but not more so than other strains that had a similar cost in glucose compared to the ancestor. This indicates that costs of constitutive expression were overcome by epistatic interactions with other mutations in the evolved background and that the reduction in cost was not due solely to a reduction in expression. Future studies will examine potential long‐term trade‐offs of compensation and what makes compensation rare.

## CONFLICT OF INTEREST

The authors declare no conflict of interest.

## AUTHOR CONTRIBUTIONS

**Kelly N. Phillips:** Conceptualization (equal); Data curation (equal); Formal analysis (equal); Investigation (equal); Methodology (equal); Software (lead); Writing‐original draft (equal); Writing‐review & editing (equal). **Tim F. Cooper:** Conceptualization (equal); Data curation (equal); Formal analysis (equal); Funding acquisition (equal); Methodology (equal); Project administration (equal); Software (supporting); Visualization (equal); Writing‐original draft (equal); Writing‐review & editing (equal).

## Supporting information

Supplementary Material

## Data Availability

T.F.C will make the strains constructed in this study available to qualified recipients following completion of an institutional material transfer agreement. The results of competition experiments, summary input data, and analysis scripts that pertain to the experiments and analyses reported in this paper have been deposited at DOI https://doi.org/10.5061/dryad.8sf7m0cp2.
