# Supplementary material for: The cost of evolved constitutive lac gene expression is usually, but not always, maintained during evolution of generalist populations
Source: Ecol Evol. 2021 Aug 6;11(18):12497–507. doi: 10.1002/ece3.7994 (PMC8462147; doi:10.1002/ece3.7994)
Supplement: Supplementary file 1 — Supplementary Material [file ECE3-11-12497-s001.docx]

**The cost of evolved constitutive *lac* gene expression is usually, but not always, maintained during evolution of generalist populations**

Kelly N. Phillips^1^, Tim F. Cooper^1,2*^

^1^ Department of Biology and Biochemistry, University of Houston, Houston, TX 77204-5001, USA

^2^ School of Natural and Computational Sciences, Massey University, Auckland 0632, New Zealand.

Table S1. Mutations occurring in sequenced clone isolated from population G/L4.

| Position | Mutation | Annotation | Gene | Description |  |
| --- | --- | --- | --- | --- | --- |
| 171,572 | Δ6 bp | coding (1247‑1252/2244 nt) | *fhuA* → | ferrichrome outer membrane transporter |  |
| 339,475 | (CCAG)_3→4_ | coding (592/1083 nt) | *lacI* ← | lac repressor | |
| 1,166,631 | G→T | D42Y (GAT→TAT) | *fabF* → | 3‑oxoacyl‑(acyl carrier protein) synthase II | |
| 1,328,333 | Δ1 bp | coding (630/1050 nt) | *sohB* → | putative periplasmic protease | |
| 1,332,148 | (T)_6→5_ | intergenic (+131/‑79) | *topA* → / → *cysB* | DNA topoisomerase I/transcriptional regulator CysB | |
| 1,619,834 | IS*150* | intergenic (‑71/+139) | *hokD* ← / ← *ECB_01533* | small toxic polypeptide/hypothetical protein | |
| 1,777,416 | T→C | K128E (AAG→GAG) | *infC* ← | translation initiation factor IF‑3 | |
| 2,199,818 | +AT | intergenic (‑48/+157) | *lysP* ← / ← *yeiE* | lysine transporter/putative DNA‑binding transcriptional regulator | |
| 2,729,858 | G→A | D363N (GAT→AAT) | *ygbD* → | nitric oxide reductase | |
| 2,871,247 | :: IS*150* | coding (28‑29/1032 nt) | *galR* → | DNA‑binding transcriptional regulator GalR | |
| 3,327,442 | +9 bp | coding (428/1104 nt) | *mreC* ← | rod shape‑determining protein MreC | |
| 3,481,558 | ::IS*1* | intergenic (‑485/‑120) | *malP* ← / → *malT* | maltodextrin phosphorylase/transcriptional regulator MalT | |
| 3,762,750 | G→A | R665H (CGC→CAC) | *spoT* → | bifunctional (p)ppGpp synthetase II/ guanosine‑3',5'‑bis pyrophosphate 3'‑pyrophosphohydrolase | |
| 3,895,023 | Δ4,352 bp | IS*150*‑mediated | *rbsD*–*[rbsK]* | *rbsD, rbsA, rbsC, rbsB, [rbsK]* | |
| 3,949,769 | (GCAGGT)_6→7_ | coding (1128/1206 nt) | *hemX* ← | putative uroporphyrinogen III C‑methyltransferase | |
| 4,100,276 | T→G | I319L (ATC→CTC) | *hslU* ← | ATP‑dependent protease ATP‑binding subunit HslU | |
| 4,329,517 | Δ5 bp | intergenic (‑768/‑1042) | *dcuR* ← / → *yjdI* | DNA‑binding transcriptional activator DcuR/hypothetical protein | |
| 4,615,696 | Δ1 bp | coding (168/1233 nt) | *nadR* → | nicotinamide‑nucleotide adenylyltransferase | |
